# Hospitalization Outcomes in Pneumocystis Pneumonia Inpatient Population: A Comparison between HIV and Non-HIV Patients

**DOI:** 10.7759/cureus.3082

**Published:** 2018-08-01

**Authors:** Sorabh Datta, Shanan Mahal, Virendrasinh Ravat, Bipin Saroha, Ehinor E Isidahome, Priya Patel

**Affiliations:** 1 Department of Emergency Medicine, New York Presbyterian Queens, Flushing, USA; 2 Department of Internal Medicine, Providence Hospital, Washington DC, USA; 3 Department of Infectious Disease, Clinical Infectious Disease Specialist, Las Vegas, USA; 4 Department of Internal Medicine, University of Chicago Medical Center, Chicago, USA; 5 Department of Internal Medicine, Saint Joseph Hospital, London, USA; 6 Department of Psychiatry, Windsor University School of Medicine, Philadelphia, USA

**Keywords:** pcp, pneumocystis pneumonia, mortality, morbidity, national inpatient sample, hospitalization, outcomes, hiv

## Abstract

Objective

To evaluate the difference in hospitalization outcomes, including morbidity and mortality among patients admitted for Pneumocystis pneumonia (PCP) with human immunodeficiency virus (HIV) and non-HIV condition.

Methods

A case-control study was done using the Healthcare Cost and Utilization Project (HCUP) Nationwide Inpatient Sample (NIS) data. We identified PCP and HIV as the primary and secondary diagnosis using ICD-9-­CM diagnosis codes. We used the multinomial logistic regression model to generate odds ratios (OR).

Results

A total number of 1250 PCP patients were enrolled in this retrospective analysis. PCP patients with HIV had eight times higher odds of non-elective admission based on emergency condition (OR = 7.873, P < .001) compared to non-HIV patients. PCP patients with HIV had eight times higher odds of longer hospitalization of more than eight days (OR = 8.687, P < .001) compared to non-HIV patients. HIV patients with PCP had five times higher odds of severe morbidity or extreme loss of body function (OR = 5.277, P < .001). PCP patients with HIV had 22 times higher likelihood of in-hospital mortality (OR = 21.845, P < .001) compared to non-HIV patients.

Conclusion

PCP patients with HIV have a higher risk of severe morbidity and in-hospital mortality as compared to non-HIV patients. More attention needs to be paid to the elderly population that is at a higher risk of PCP with HIV. We need additional research and studies to direct the development of clinical care models for aiming early diagnosis and treatment of HIV in PCP patients.

## Introduction

Pneumocystis pneumonia (PCP) is an opportunistic infection of the lung caused by the fungus Pneumocystis jirovecii [1]. PCP is commonly seen in immunocompromised individuals [2].

PCP is also a common opportunistic infection among people living with human immunodeficiency virus/acquired immunodeficiency syndrome (HIV/AIDS) in developing countries [3]. HIV-infected patients with a low CD4 count are at the highest risk of PCP. Others at substantial risk include hematopoietic cell and solid organ transplant recipients, those with cancer (particularly hematologic malignancies), and those receiving glucocorticoids, chemotherapeutic agents, and other immunosuppressive medications.

The incidence of PCP is increasing as the number of people receiving immunosuppressive medications continues to grow [4]. The incidence of PCP appears to vary by underlying condition, as in hematologic malignancies it is 32.5%, 18.2% in solid tumors, 14.9% in inflammatory diseases, and 12.3% in solid organ transplant [5]. In patients with malignancies, the reported rates vary with underlying disease and immunosuppressive regimen, it is 22%–45% in children with acute lymphoblastic leukemia without prophylaxis, which decreases to 0% with prophylaxis, 25% among patients with Hodgkin lymphoma without prophylaxis and 1.3% among patients with primary or metastatic central nervous system (CNS) tumor with available prophylaxis [4, 6]. Also, there was 1% to 2% incidence in patients with rheumatologic disorders who were not taking prophylaxis treatment, especially those patients which were receiving immunosuppressive medications [4]. The mortality rate is about 30–50% in patients without HIV [7, 8] and it is around 10–12% in patients with HIV [9].

The main objective of this study is to analyze the differences in hospitalization outcomes in terms of hospital stay and cost, morbidity and mortality in PCP patients with HIV versus with non-HIV conditions.

## Materials and methods

Data source

The Agency for Healthcare Research and Quality (AHRQ) sponsors the Healthcare Cost and Utilization Project (HCUP) databases that are specifically designed to determine and identify patterns in utilization and cost across the United States hospitals [10]. The Nationwide Inpatient Sample (NIS) database is the inpatient database available in the United States. Any information about the patients, physicians, hospitals, and hospital identifiers is de-identified, to protect the privacy of the individual. This data contains some non-clinical information of patient’s demographic data, hospital characteristics, and inpatient charges, and the clinical-related information includes primary, and secondary diagnosis, disposition or discharge status and the length of inpatient stay.

Variables of interest

Based on the International Classification of Diseases, 9th Edition, Clinical Modifications (ICD-9-CM) diagnosis codes, we identified the controls that included patients with a primary diagnosis of PCP during hospital admission and a secondary diagnosis of the non-HIV conditions as mentioned in Table 1. The cases were identified with a primary diagnosis of PCP and secondary diagnosis of HIV. PCP was identified using diagnosis code 136.3, and HIV was identified using ICD-9-CM diagnosis codes 042, 0420-0422, 0429-0433, 0439, 0440, 0449 079.53, 279.10, 279.19, 795.71, 795.8 or V08. To measure the differences in hospitalization outcomes in PCP patients with HIV versus PCP with the non-HIV condition, the outcome variables of interest included the severity of illness, inpatient length of stay, inpatient total charges, disposition of patient and in-hospital mortality [11].

**Table 1:** ICD-9-CM diagnosis codes for non-HIV conditions. ICD-9-CM: International Classification of Diseases, 9th Revision, Clinical Modification

Disease	ICD-9-CM Diagnosis Code
Hodgkin’s Lymphoma	201.00-201.18, 201.20-201.28, 201.40-201.48, 201.50-201.58, 201.60-201.68, 201.70-201.78, 201.90-201.98, V10.72, 202.21-202.28, 202.70-202.78, 202.80-202.88, 202.90-202.98, V10.71, V10.79
Non-Hodgkin’s Lymphoma	200.00-200.08, 200.10-200.18, 200.20-200.28, 200.30-200.38, 200.40-200.48, 200.50-200.58, 200.60-200.68, 200.70-200.78, 200.80-200.88, 202.00-202.08, 202.10-202.18, 202.20
	202.40-202.48, 203.1, 203.10-203.12, 204.0, 204.00-204.02, 204.1, 204.10- 204.12, 204.2, 204.20-204.22, 204.8, 204.80-204.82, 204.9, 204.90-204.92, 205.0, 205.00-205.02, 205.1, 205.10-205.12, 205.2, 205.20-205.22, 205.3, 205.30-205.32, 205.8, 205.80-205.82, 205.9, 205.90-205.92, 206.0, 206.00-206.02, 206.1, 206.10-20612, 206.2, 206.20-206.22, 206.8, 206.80-206.82, 206.9, 206.90-206.92, 207.0, 207.00-207.02, 207.1, 207.10-207.12, 207.2, 207.20-207.22, 207.8, 207.80-207.82, 208.0, 208.00-208.02, 208.1, 208.10-208.12, 208.2, 208.20-208.22, 208.8, 208.80-208.82, 208.9, 208.90-208.92, V10.60-V10.63, V10.69
Common Variable Immunodeficiency	279.06, 279.00, 279.05, 279.3
Familial Hemophagocytic lymphohistiocytosis	288.4, D76.1
X-Linked agammaglobulinemia	279.04, D80.0
Pancytopenia	284.19, 284.09, 284.11, 284.12
Chronic Granulomatous disease	288.1, 686.1
Wiskott-Aldrich Syndrome	279.12, 279.3, 279.10, 279.2, 279.19
Neutropenia	780.60, 288.00-288.04, 288.09, 776.7, 289.53
Chronic lymphocytic leukemia	204.10
Complement Deficiency Asplenia	279.8
Multiple Myeloma	203.0, 203.00-203.02, 203.8, 203.80-203.82

Case and control selection

Cases were selected from the NIS dataset with a primary diagnosis of PCP, the secondary diagnosis of HIV and age >18 years. Controls included the patients with a primary diagnosis of PCP, a secondary diagnosis of non-HIV condition as mentioned in Table 1 and were matched with the cases for age, gender and race.

Statistical analysis

A cross-sectional study was conducted using the HCUP NIS data from 2012 to 2014. An exploratory data analysis using cross tabulation was performed on the NIS database, thereby targeting the PCP patients with HIV versus with non-HIV conditions. Pearson’s Chi-square test and independent sample T-test were used for categorical data and continuous data, respectively. Multinomial logistic regression model for differences in hospital outcomes was used to calculate adjusted odds ratio (aOR) and was adjusted for age, gender, and the race. All the tests were two-sided and a p-value < .05 was used as a referent for a statistical significance test result. The analysis was conducted using Statistical Package for the Social Sciences (IBM, Armonk, NY) [12]. The study database does not contain any patient identification and so we were not required to take the Institution Review Board (IRB) permission for this study.

## Results

Demographic characteristics

We analyzed a total number of 1250 PCP patients in this study from which 895 cases had HIV and 355 controls had non-HIV conditions. PCP with HIV was seen more in older patients aged 51–60 years (N = 230; 25.7%), and was equally common in 41–50 years (N = 155; 17.3%) and 61–70 years’ age (N = 160; 17.9%) patients. PCP is seen in a low proportion of young patients with HIV (7.8% in 18–30 years and 9.5% in 31–40 years’ age). Majority of the HIV patients were males (N = 525; 58.7%) and Caucasians (N = 450; 53.3%). The demographic distribution of the sample population is mentioned in Table 2.

**Table 2:** Demographic distribution in PCP patients by HIV. PCP: Pneumocystis carinii pneumonia

Variable	Non-HIV		HIV	
	N	%	N	%
Age				
18-30 years	5	1.4	70	7.8
31-40 years	20	5.6	85	9.5
41-50 years	60	16.9	155	17.3
51-60 years	85	23.9	230	25.7
61-70 years	85	23.9	160	17.9
>70 years	100	28.2	195	21.8
Gender				
Male	225	63.4	525	58.7
Female	130	36.6	370	41.3
Race				
Caucasian	275	77.5	450	53.3
African American	65	18.3	210	24.9
Hispanic	10	2.8	100	11.8
Asian/Pacific Islander	5	1.4	30	3.6
Native American	0	0	5	.6
Other	0	0	50	5.9

Differences in hospitalization outcomes

About 93.9% (N = 840) PCP patients with HIV were admitted based on an emergency condition or non-elective basis, whereas 91.5% (N = 325) were admitted on an elective basis. PCP patients with HIV had eight times higher odds of non-elective admission based on emergency condition (OR = 7.873, p < .001) compared to non-HIV patients. Also, HIV patients had higher odds of being transferred from another health facility (OR = 13.646, p < .001) followed by acute care hospital (OR = 10.701, p < .001) compared to non-HIV patients.

The mean inpatient length of stay per admission for PCP patients with HIV was 11.78 days which was marginally higher than that seen in non-HIV patients (10.1 days). The median inpatient length of stay per admission for all PCP patients was eight days and 48.6% (N = 435) HIV patients were hospitalized for more than eight days as compared to 47.9% (N = 170) non-HIV patients, though the result was not statistically significant (p = .819). However, HIV patients had eight times higher odds of longer hospitalization of more than eight days (OR = 8.687, p < .001) compared to non-HIV patients with PCP. The median hospitalization cost per admission for PCP was $59,923. PCP patients with HIV had ten times likelihood of higher hospitalization charges of more than $59,923 (OR = 10.229, p < .001) compared to non-HIV patients.

HIV patients with PCP had five times higher odds of severe morbidity or major loss of body function (OR = 5.851, p < .001) compared to non-HIV patients. Also, 18.4% (N = 165) PCP patients with HIV died during hospitalization compared to 11.3% (N = 40) patients with non-HIV conditions (P = .002). HIV patients with PCP had 22 times higher odds of in-hospital mortality (OR = 21.845, p < .001) compared to non-HIV patients. Differences in hospitalization outcomes are shown in Table 3.

**Table 3:** Hospital outcomes in PCP patients by HIV. Significant p ≤ 0.05 at 95% confidence interval. PCP: Pneumocystis carinii pneumonia

Variable	Non-HIV		HIV		P
	N	%	N	%	
Admission Type					
Non-elective	325	91.5	840	93.9	.144
Elective	30	8.5	55	6.1	
Transfer from facility					
Acute care hospital	15	4.2	50	5.6	.579
Another health facility	5	1.4	15	1.7	
Severity of illness or morbidity					
Moderate loss of body function	0	0	95	10.6	<.001
Major loss of body function	355	100	800	89.4	
Inpatient length of stay per admission					
Mean	10.1 days		11.78 days		.008
>8 days (median)	170	47.9	435	48.6	.819
Inpatient total cost per admission					
Mean	$93,305		$106,752		.104
>$59,923 (median)	165	46.5	450	50.3	.226
In-hospital mortality					
Deaths during hospitalization	40	11.3	165	18.4	.002
Disposition of patient					
Routine	210	59.2	440	49.2	.012
Short-term hospital	5	1.4	20	2.2	
Skilled nursing/intermediate nursing facility	60	16.9	160	17.9	
Home health care	35	9.9	90	10.1	
Against medical advice	5	1.4	20	2.2	

A higher proportion of HIV patients (N = 160; 17.9%) were discharged to skilled nursing facility or intermediate nursing facility (SNF/INF), and so they had very high odds of discharge to SNF/INF (OR = 23.238, p < .001) compared to non-HIV patients (N = 60; 16.9%). Also, HIV patients had a higher likelihood of disposition to the short-term hospital (OR = 22.230, p < .001) compared to non-HIV patients. Association of adverse hospitalization outcomes in HIV patients during admission for PCP is mentioned in Table 4.

**Table 4:** Association of adverse hospital outcomes in PCP with HIV patients. Significant p ≤ .05 at 95% confidence interval, Odds Ratio were adjusted for age, gender and race. PCP: Pneumocystis carinii pneumonia; SNF: Skilled nursing facility; INF: Intermediate nursing facility

Variable	Odds Ratio	95% Confidence Interval		P
		Lower Bound	Upper Bound	
Transfer from acute care hospital	10.701	4.908	23.333	< .001
Transfer from another health facility	13.646	4.261	43.705	< .001
Non-elective/Emergency admission	7.873	4.742	13.071	< .001
Inpatient length of stay > 8 days	8.687	5.041	14.971	< .001
Inpatient total cost > $59,923	10.229	5.851	17.884	< .001
Major loss of body function/morbidity	5.277	3.144	8.858	< .001
In-hospital mortality	21.845	11.012	43.337	< .001
Disposition to SNF/INF	23.238	11.107	48.617	< .001
Disposition to short-term hospital	22.230	7.083	69.770	< .001

## Discussion

In this study, among older patients above 60 years’ age, PCP was more prevalent in non-HIV patients as compared to HIV patients, which could be due to the fact that older patients were less likely to have acquired HIV via intravenous drug use or homosexual contact as compared to patients below 60 years. Also, older patients were more likely to have comorbid diseases (non-HIV conditions) like cancer, end-stage renal disease and ageing [13, 14]. Among 41–50 years and 51–60 years aged patients, PCP was more common in HIV patients. This is due to an increase in the number of HIV diagnoses among males aged 18–60 years which was driven by an increase in HIV diagnoses among young homosexual men [15]. Also based on the previous studies, HIV is more prevalent in the age group 18–60 years as compared to >60 years age patients [16]. In our study, the majority of the PCP patients with HIV were males as compared to females, it could be due to the fact that females may be at higher epidemiologic and socio-behavioral risk for HIV. Also, they lack early counseling, diagnosis and treatment as compared to males [17]. Males, however, were better informed about the use of Antiretrovirals than females and also due to the fact that women had a lower socioeconomic status or to some biological differences as compared to men [18].

PCP patients with HIV had much higher admission rates based on emergency condition compared to non-HIV patients in this study. Majority of the PCP patients with HIV are coming with acute symptoms like an acute respiratory failure, which is a complication of PCP in 5%–30% of cases and is associated with a high risk of in-hospital death [19]. PCP is a major cause of life-threatening pneumonia in the immunocompromised host, therefore they are more presented as an emergency case in PCP patients with HIV [20]. In the previous study, the pooled overall mortality for non-HIV patients with PCP was significantly higher than previously reported mortality rate in HIV patients. There are several possible explanations for the poorer outcomes in non-HIV patients with PCP such as the majority of these patients were older and had the more underlying cardiopulmonary disease than HIV-positive patients with PCP. The duration of symptoms onset to the beginning of PCP treatment was much longer in non-HIV patients. Also, HIV patients with PCP were benefited from adjunctive corticosteroid therapy, but there is no proof that adjunctive corticosteroid is beneficial to non-HIV patients. This suggests that non-HIV PCP may not benefit from the advances in the management of PCP [21]. In the current study, PCP patients with HIV were having a longer inpatient length of stay as compared to non-HIV patients. This fact can be supported by previous studies where the duration of symptoms in HIV-infected patients was consistently longer than for HIV-negative patients [22-24].

In our study, morbidity and mortality are much higher in HIV patients with PCP as compared to non-HIV patients with PCP. And it can be supported with past studies which show that due to the use of PCP prophylaxis the incidence of PCP is low, but that PCP is still a common disease in patients unaware of their HIV infection. On the other hand, a study also suggests that CNS disease is the major immediate cause of death in patients on PCP prophylaxis [25]. Based on various studies we also found that Highly Active Antiretroviral Treatment (HAART) is independently and statistically significantly associated with decreased mortality and morbidity rates in PCP patients [26].

To identify the significant strength of our study, a nationally represented dataset was selected with the inclusion of a uniform collection of data through ICD-9-CM codes. To our knowledge, this is the first study to report the impact of HIV on PCP patients regarding hospital outcomes, morbidity, and mortality. The NIS dataset is subject to a minimal reporting bias. Also, all the information from this dataset is coded independently of the practitioner, which makes it a dependable source.

Limitations of our study include the lack of patient-level data from the NIS dataset, which is needed to make accurate clinical associations. As an administrative database, the NIS dataset has this limitation. Besides, such retrospective studies are always subject to selection bias, which might be highlighted by the moderate sensitivity of diagnostic codes for PCP, HIV and non-HIV conditions. After considering all the potential limitations with overall comparisons, the NIS database presents an unparalleled population-based perspective on disease associations with systematic and temporal factors providing a rationale for further in-depth studies.

## Conclusions

Among PCP patients with HIV versus without HIV, we observed variation in demographic and hospital characteristics and significant differences in the risk of in-hospital mortality. Men with PCP were more prevalent in the non-HIV group as compared to female patients with PCP which were more prevalent in the HIV group. Also, PCP patients with HIV have greater all-cause mortality as compared to patients without HIV. From this study, we also see that there is a rise in PCP among women with the comorbid HIV which needs to be addressed with educational training regarding primary and secondary prevention of HIV. To decrease the HIV-related mortality and morbidity in these patients, the future direction for PCP management should include strategies for early diagnosis and treatment of HIV.
